# The process of risky sexual behaviors formation in women drug users: a protocol for a grounded theory study

**DOI:** 10.1186/s12978-020-00936-5

**Published:** 2020-06-01

**Authors:** Hadis Sourinejad, Mahnaz Noroozi, Fariba Taleghani, Gholam Reza Kheirabadi

**Affiliations:** 1 Student Research Committee, School of Nursing and Midwifery, Isfahan University of Medical Sciences, Isfahan, Iran; 2 Department of Midwifery and Reproductive Health, School of Nursing and Midwifery, Isfahan University of Medical Sciences, Isfahan, Iran; 3 Department of Adult Health Nursing, School of Nursing & Midwifery, Isfahan University of Medical Sciences, Isfahan, Iran; 4 Behavioral Sciences Research Center, Department of Psychiatry, School of Medicine, Isfahan University of Medical Sciences, Isfahan, Iran

**Keywords:** Sexual behavior, Unsafe sex, Drug users, Substance use disorders, Women, Grounded theory

## Abstract

**Background:**

Drug use is an important underlying factor in risky sexual behaviors. Risky sexual behaviors can lead to STIs and HIV/AIDS, especially in women. For better understanding of the relationship between drug use and risky sexual behaviors in women, it is necessary to identify the process of the formation of these behaviors that is a multidimensional process influenced by multiple socio-cultural factors. Therefore, the present study aims to explore the process of risky sexual behaviors formation in women drug users.

**Methods:**

This is a grounded theory qualitative study with Corbin and Strauss approach. The participants of the study are women drug users with risky sexual behaviors who, using purposeful sampling method, will be selected from the Counseling and Harm Reduction centers for vulnerable women, the Drug Rehabilitation centers affiliated to the Isfahan University of Medical Sciences, Therapeutic Community Rehabilitation centers, Drop in Centers affiliated to the Welfare Organization, Medium-term Residential centers (women’s camps), and Women’s Empowerment centers in Isfahan, Iran. Sampling will continue using snowball method and the strategy of maximum variation in terms of the age, occupation, education, duration of the drug use, and type of the drug. During the sampling process, theoretical sampling will gradually replace purposeful sampling, so that sampling will proceed based on the emergence of the theory and for understanding of the concept and, then, the next participants will be selected. Sampling will continue until data saturation is reached. Data will be collected using individual semi-structured in-depth interviews, observation, field notes, and memo writing. Data will be coded as they are collected, and the analysis will be performed at three levels of open, axial, and selective coding and based on the constant comparative analysis. The four criteria of credibility, dependability, transferability and confirmability will be used to ensure the trustworthiness of the data.

**Discussion:**

The findings of the present study are expected to provide a better understanding of the process of risky sexual behaviors formation in women drug users. The findings may also lead to the identification of the barriers and factors contributing to the formation of such behaviors and, finally, will promote the reproductive and sexual health of these women. This study can also provide the guide and the ground for designing and conducting further studies in the related areas through using various qualitative and quantitative methods.

## Plain English summary

The staggering increase of drug use in the world together with the increased drug trafficking has undesirable consequences that threaten humanity, particularly the younger generation, in the social, economic, cultural and political dimensions. Drug use has been mentioned as an important underlying factor in the formation of the self-harm and high-risk behaviors. Among high-risk behaviors, risky sexual behaviors have been more emphasized as the most prominent human concerns that is due to their adverse and irreversible consequences such as the incidence of STIs and HIV/AIDS. The biological effects and consequences, the progress and occurrence of drug-related disorders vary significantly between men and women, that is, they are usually more severe in women. Given the increasing prevalence of drug use among women, this study aims to explore the process of risky sexual behaviors formation among women drug users in the social context of Isfahan, Iran. The participants of this study are women drug users with risky sexual behaviors who will be selected using purposeful sampling method. Sampling will continue using snowball sampling method and the strategy of maximum variation in terms of the age, occupation, education, duration of the drug use, and type of the drug. During the sampling process, theoretical sampling will gradually replace purposeful sampling method. Accordingly, the next participants will be selected considering what specific data is needed following the initial findings. Data will be collected using semi-structured in-depth interviews, observation, field notes, and memo writing. Along with data collection, they will be coded and analyzed to obtain a theory-based data based on the constant comparative analysis. The results of the present study are expected to provide an in-depth understanding of the process of risky sexual behaviors formation, barriers and the factors reinforcing these behaviors in women drug users. The results of this study can be used for planning and health policy making in this area.

## Background

Drug use is considered as the most significant social harm which has affected human societies [1, 2]. Through its devastating effects on human health, crime and mortality, this issue has imposed enormous social and economic costs on societies and has, thus, become a major threat to human societies [3]. According to the latest UN report in 2019, about 280 million people worldwide use drugs [4]. According to one of the most recent studies conducted in Iran in 2016, the 12-month prevalence of illicit drug use, based on the DSM-IV and DSM-V criteria, was reported to be 2.1 and 2.4% respectively, suggesting that there are 1.12 million drug users among Iranian adults [5]. There are currently no accurate statistics about the number of women drug users in Iran, but according to the Ministry of Health and Medical Education, the ratio of the female drug user to the male ones is one to eight [6]. According to the results of the previous studies, there are remarkable differences between men and women in terms of the process of drug use initiation, social factors and features related to drug use, biological effects and consequences, and the progress and occurrence of substance-related disorders [7–9]. Drug use has been mentioned as an important underlying factor in the formation of self-harm and high-risk behaviors [10]. Because of their adverse and irreversible consequences such as sexually transmitted infections (STIs), HIV/AIDS, increased risk of cervical cancer and unwanted pregnancy, risky sexual behaviors have recently been emphasized more than other risky behaviors [11, 12]. Risky sexual behaviors are more prevalent among drug users than the general population, and according to the reports the type of drug has little effect on the relationship between drug use and the occurrence of risky sexual behaviors [13]. Studies in Iran show that about 55% of drug users have out-of-wedlock relationships, indicating a high prevalence of having multiple sex partners in this group [14, 15]. It should be noted that women, because of their sensitive nature, are more affected by the deleterious consequences of risky sexual behaviors [16]. Ritchwood’s study has also reported that drug use in women is more strongly associated with risky sexual behaviors than men [13].

Although the prevalence of drug use in women is increasing as in men [17, 18], there is little information about drug use and its consequences in Iran, and the extent and nature of it in women is less known [19]. It is believed that solutions to this problem vary based on the social context and conditions of each region, at-risk individuals and risk factors; as such, there are many cultural differences in this regard [20, 21]. In this regard, grounded theory study is a kind of qualitative study leading to a theory derived from data on the characteristics and actual conditions of the research subject in its social context and environment [22]. Given the human, social, interactive, and procedural nature of risky sexual behaviors in women drug users, it seems necessary to conduct a grounded theory study in order to explore and identify the process of risky sexual behaviors of women drug users in the social context.

## Objective

The discovery of the process of risky sexual behaviors formation in women drug uses.

## Methods/ design

This study is a grounded theory qualitative study which uses Corbin and Strauss approach [23].

### Settings, sample and recruitment

The participants of the study will be women drug users with risky sexual behaviors who live in Isfahan, Iran. These women will be selected from the Counseling and Harm Reduction centers for vulnerable women, the Drug Rehabilitation centers affiliated to the Isfahan University of Medical Sciences, Therapeutic Community Rehabilitation centers, Drop in Centers (DIC) affiliated to the Welfare Organization, Medium-term Residential centers (women’s camps), and Women’s Empowerment centers. In the present study, sampling will be started using purposeful sampling method and will be continued using snowball method and the strategy of maximum variation in terms of the age, occupation, education, duration of the drug use, and type of the drug. During the research process, theoretical sampling will gradually replace purposeful sampling, so that sampling will proceed based on the emergence of the theory and for understanding of the concept. Thus, the next participants will be selected considering what specific data is needed following the initial findings. In fact, who will be selected later depends on those who have been selected before and the information they have provided. In the present study, sampling will continue until data saturation is reached, that is, until the researcher determines that the main categories are sufficiently developed in terms of the depth and variability as well as the relationship between the categories is clear.

### Inclusion criteria

Women with definitive diagnosis of substance use disorders (SUD), (diagnosis will be based on the criteria in the DSM-5 classification); women over 18 years of age who have initiated risky sexual behaviors after being engaged in substance use disorders; tendency to participate in the study and having informed consent for providing the necessary information; ability to communicate and conduct interviews; Iranian citizenship and ability to speak Persian.

### Data collection process

In this study, individual in-depth and semi-structured interviews will be used for data collection. Interviews will be recorded using a tape recorder. During the interviews, considerations will be made to create a safe and confidential environment for the participants so that they can express their experiences and views more confidently. Some of the interview guide questions are as follows: How did you start using drugs? Please explain it? How did you get into sexual relationships with different people? Please explain. What made you continue to have sex with different people? Could you explain please? As the research process continues and in order to achieve the research objectives, more specific questions will be asked in the interviews based on the main themes and categories of the previous interviews. During the interviews, non-verbal behaviors of the participants will be considered and recorded by the researcher as field notes.

In the present study, the observation method will also be used for data collection; so that the researcher observes the interaction of these women with each other and with others (addiction therapists, service provider personnel, etc.) and records their verbal behaviors, nonverbal behaviors, and their emotional reactions in the form of field notes. In the present study, the researcher’s thoughts and interpretations will be recorded as memo while analyzing the data. Memos will help the researcher understand what is in the data by describing the concepts and finding the relationship between them, and interpret the results using the data. Memos will also, in some cases, help the researcher what questions to ask, where to look for data, and how to complete the data for the next interviews.

### Data analysis

For data analysis, the constant comparative analysis with Corbin and Strauss approach [23] will be used. The researcher collects, encodes, and analyzes the data simultaneously. To this end, all interviews will be read line-by-line, revised repeatedly, and coded as open, axial, and selective coding. As such, following the constant comparison of codes in terms of the similarities and differences in concepts and themes, categories will be formed.

### Rigor and trustworthiness

The criteria proposed by Lincoln and Guba [24], including credibility, transferability, dependability, and confirmability will be used to assess the rigor and trustworthiness of the data. To maintain the rigor of the data, various methods including in-depth interviews at different places and times, combining different methods of data collection such as personal interviews, field noting and memoing; and selecting the participants with maximum variation (regarding their age, educational level, occupation, type of substance used and duration of substance use) will be applied. In this study, transcripts will be returned to participants for comments or corrections. Also, to confirm the validity of the data, during other sessions, the coded interviews were given to the participants and their final opinions will be achieved; therefore member check will be obtained. The opinions of the experts will be obtained to assure the consistency of the results. In the present study, to enhance the transferability of the data, results of the study will be given to the individuals with similar characteristics to the participants who did not participate in the study to judge the similarity of the results to their own experiences.

### Ethical considerations

This present study was approved by the Ethics Committee of Isfahan University of Medical Sciences (ethics code: IR.MUI.RESEACH.REC.1398.667). The reasons for the study will be explained prior to each individual interview. Also, obtaining informed consent, confidentiality of the data, maintaining anonymity and the right of withdrawal at any desired time will be practiced.

## Discussion

The issue of drug use and its association with risky sexual behaviors is a multifaceted issue that requires the intervention of experts in various fields and institutions, and intra-organizational planning alone is not sufficient for a comprehensive look at it [25–27]. The focus of the present study is on two major issues: first, the issue of drug use among women that, according to reports, is increasing as quickly as it is increasing among men [28, 29]. Although this issue is more veiled in Iran because of its cultural context, according to existing studies and evidence, drug use, as an important social problem, has become a major issue for women [29]. The second issue is related to the risky sexual behaviors that cause many issues, problems, costs and financial burdens. In this regard, drug use has been described as the strongest cause and predictor of exposure to gonorrhea, herpes simplex virus (HSV), and HIV [30–32]. Since risky sexual behaviors in women drug users can be shaped based on their perceptions of social interactions, this grounded theory study will be conducted for understanding and identification of the process of risky sexual behaviors in their natural environment and social context. Grounded theory studies are the best ones for doing explanatory research about the phenomena of which our knowledge is still limited and we want to get a better understanding of them [33]. These studies identify processes in their social context and analyze, define and interpret process-related factors and situations; they also describe and explain interactive processes between individuals and groups in a social context [22]. Given that the process leading to the creation of risky sexual behaviors in women drug users is a multidimensional process influenced by multiple socio-cultural factors, and as this issue, because of the cultural context of Iran, has a more hidden form in women than men [34], it seems that the investigation of the process of risky sexual behaviors formation in women drug users through a grounded theory study can lead to a better understanding of the relationship between drug use and risky sexual behaviors. The findings of this study are expected to contribute to the advent of a theory in the area of understanding the process of risky sexual behaviors in women drug users, as well as the identification and reinforcement of the factors and barriers to the creation of these behaviors. Doing so, the findings can provide the country’s health policy makers and planners with useful information for reducing risky sexual behaviors and their complications in women drug users, thereby promoting these women’s sexual and reproductive health. Finally, the results of this study can be used as a guide and starting point for designing and conducting future studies in related areas and using different quantitative and qualitative methods.

## Data Availability

Not applicable.

## Abbreviations

STIs: Sexually Transmitted Infections
HIV: Human Immunodeficiency virus
AIDS: Acquired Immune Deficiency Syndrome
DSM: Diagnostic and Statistical Manual of Mental Disorders
SUD: Substance Use Disorder
HSV: Herpes Simplex Virus
